# 2,3-[(3,6-Dioxaoctane-1,8-diyl)bis(sul­fanediylmethylene)]-6,7-bis(methylsulfanyl)-1,4,5,8-tetra­thia­fulvalene

**DOI:** 10.1107/S1600536809041804

**Published:** 2009-10-17

**Authors:** Rui-Bin Hou, Bao Li, Tie Chen, Bing-Zhu Yin, Li-Xin Wu

**Affiliations:** a Key Laboratory of Organism Functional Factors of Changbai Moutain, Yanbian University, Ministry of Education, Yanji 133002, People’s Republic of China; b State Key Laboratory of Supramolecular Structure and Materials, College of Chemistry, Jilin University, Changchun 130012, People’s Republic of China

## Abstract

In the title mol­ecule, C_16_H_22_S_8_O_2_, two S atoms, two O atoms and ten C atoms form a 14-membered ring with a boat conformation. In the crystal, C—H⋯O hydrogen bonds link the mol­ecules into dimers which are further connected into a chain along the *a* axis by C—H⋯S hydrogen bonds.

## Related literature

Over the past three decades, chemical groups such as crown ethers have been extensively modified on the tetra­thia­fulvalene (TTF) skeleton, see: Jeppesen & Becher (2003 ▶). For details of the synthesis, see: Chen *et al.* (2005 ▶). For a related structure, see: Hou *et al.* (2009 ▶).
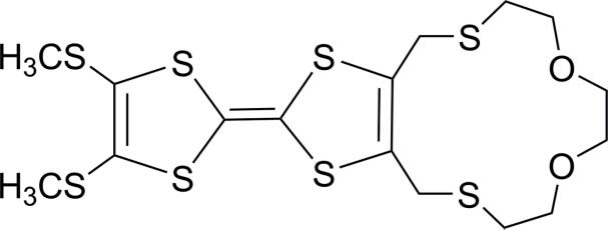


## Experimental

### 

#### Crystal data

C_16_H_22_O_2_S_8_
*M* *_r_* = 502.82
Triclinic, 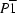

*a* = 9.1748 (18) Å
*b* = 10.177 (2) Å
*c* = 14.273 (3) Å
α = 98.49 (3)°
β = 105.58 (3)°
γ = 113.33 (3)°
*V* = 1129.1 (4) Å^3^
*Z* = 2
Mo *K*α radiation
μ = 0.80 mm^−1^
*T* = 291 K
0.14 × 0.12 × 0.12 mm

#### Data collection

Rigaku R-AXIS RAPID diffractometer
Absorption correction: multi-scan (*ABSCOR*; Higashi, 1995 ▶) *T*_min_ = 0.896, *T*_max_ = 0.910
8752 measured reflections
3948 independent reflections
3345 reflections with *I* > 2σ(*I*)
*R*_int_ = 0.014

#### Refinement

*R*[*F* ^2^ > 2σ(*F* ^2^)] = 0.042
*wR*(*F* ^2^) = 0.128
*S* = 1.17
3948 reflections
237 parameters
H-atom parameters constrained
Δρ_max_ = 0.89 e Å^−3^
Δρ_min_ = −0.47 e Å^−3^

### 

Data collection: *RAPID-AUTO* (Rigaku, 1998 ▶); cell refinement: *RAPID-AUTO*; data reduction: *CrystalStructure* (Rigaku/MSC and Rigaku, 2002 ▶); program(s) used to solve structure: *SHELXS97* (Sheldrick, 2008 ▶); program(s) used to refine structure: *SHELXL97* (Sheldrick, 2008 ▶); molecular graphics: *PLATON* (Spek, 2009 ▶); software used to prepare material for publication: *SHELXL97*.

## Supplementary Material

Crystal structure: contains datablocks global, I. DOI: 10.1107/S1600536809041804/hg2569sup1.cif

Structure factors: contains datablocks I. DOI: 10.1107/S1600536809041804/hg2569Isup2.hkl

Additional supplementary materials:  crystallographic information; 3D view; checkCIF report

## Figures and Tables

**Table 1:** Hydrogen-bond geometry (Å, °)

*D*—H⋯*A*	*D*—H	H⋯*A*	*D*⋯*A*	*D*—H⋯*A*
C8—H8*A*⋯S5^i^	0.97	2.94	3.762 (5)	143
C1—H1*C*⋯O2^ii^	0.96	2.49	3.379 (6)	154

Symmetry codes: (i) 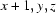; (ii) 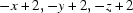.

**Fig. 1.**
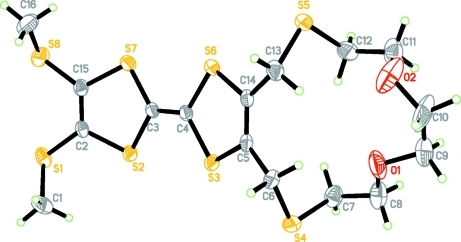
The asymmetric of title compound, with the atom numbering. Displacement ellipsoids of non-H atoms are drawn at the 30% probability level.

## supplementary crystallographic information

### Comment

Over the past three decades, chemical groups such as crown ethers have been extensively modified on the tetrathiafulvalene (TTF) skeleton (Jeppesen *et al.*, 2003). A series of different type and different ring size TTF crown ether derivatives (containing nitrogen, sulfur and thia-aza atoms), aiming at molecular sensors, switches and wires in which TTF was used as an organic redox-active unit in host–guest system and crown ether was used as the choice of ligand system suitable as an "antenna". In order to get a novel and promising ion sensor, we have designed and synthesized the TTF - crown ether title compound, (I).

In the structure (I) (Fig. 1) all bond lengths and angles are normal and comparable with those reported for the related structure (Hou *et al.*, 2009). In the crystal lattice, two molecules form a dimer by C—H···O hydrogen bonds, involving one O atom of the crown ether as acceptors, and the methylene C—H groups as donors (Table 1). The intermolecular C—H···S hydrogen bonds link the dimers into one-dimensional chain along the *a* axis.

### Experimental

The title compound, (I), was prepared according to literature (Chen *et al.*, 2005) and single crystals suitable for X-ray diffraction were prepared by slow evaporation a mixture of dichloromethane and petroleum (60–90°C) at room temperature.

### Refinement

Carbon-bound H-atoms were placed in calculated positions (C—H 0.96-0.97Å) and were included in the refinement in the riding model with *U*_iso_(H) = 1.2 or 1.5 *U*_eq_(C).

### Crystal data

**Table:**

C_16_H_22_O_2_S_8_	*Z* = 2
*M**_r_* = 502.82	*F*(000) = 524
Triclinic, *P*1	*D*_x_ = 1.479 Mg m^−^^3^
Hall symbol: -P 1	Mo *K*α radiation, λ = 0.71073 Å
*a* = 9.1748 (18) Å	Cell parameters from 9031 reflections
*b* = 10.177 (2) Å	θ = 3.1–27.5°
*c* = 14.273 (3) Å	µ = 0.80 mm^−^^1^
α = 98.49 (3)°	*T* = 291 K
β = 105.58 (3)°	Block, yellow
γ = 113.33 (3)°	0.14 × 0.12 × 0.12 mm
*V* = 1129.1 (4) Å^3^	

### Data collection

**Table:**

Rigaku R-AXIS RAPID diffractometer	3948 independent reflections
Radiation source: fine-focus sealed tube	3345 reflections with *I* > 2σ(*I*)
graphite	*R*_int_ = 0.014
ω scans	θ_max_ = 25.0°, θ_min_ = 3.1°
Absorption correction: multi-scan (*ABSCOR*; Higashi, 1995)	*h* = −10→10
*T*_min_ = 0.896, *T*_max_ = 0.910	*k* = −12→12
8752 measured reflections	*l* = −16→15

### Refinement

**Table:**

Refinement on *F*^2^	Primary atom site location: structure-invariant direct methods
Least-squares matrix: full	Secondary atom site location: difference Fourier map
*R*[*F*^2^ > 2σ(*F*^2^)] = 0.042	Hydrogen site location: inferred from neighbouring sites
*wR*(*F*^2^) = 0.128	H-atom parameters constrained
*S* = 1.17	*w* = 1/[σ^2^(*F*_o_^2^) + (0.0734*P*)^2^ + 0.3957*P*] where *P* = (*F*_o_^2^ + 2*F*_c_^2^)/3
3948 reflections	(Δ/σ)_max_ = 0.011
237 parameters	Δρ_max_ = 0.89 e Å^−^^3^
0 restraints	Δρ_min_ = −0.47 e Å^−^^3^

### Special details

**Table:**

Experimental. (See detailed section in the paper)
Geometry. All e.s.d.'s (except the e.s.d. in the dihedral angle between two l.s. planes) are estimated using the full covariance matrix. The cell e.s.d.'s are taken into account individually in the estimation of e.s.d.'s in distances, angles and torsion angles; correlations between e.s.d.'s in cell parameters are only used when they are defined by crystal symmetry. An approximate (isotropic) treatment of cell e.s.d.'s is used for estimating e.s.d.'s involving l.s. planes.
Refinement. Refinement of *F*^2^ against ALL reflections. The weighted *R*-factor *wR* and goodness of fit *S* are based on *F*^2^, conventional *R*-factors *R* are based on *F*, with *F* set to zero for negative *F*^2^. The threshold expression of *F*^2^ > σ(*F*^2^) is used only for calculating *R*-factors(gt) *etc*. and is not relevant to the choice of reflections for refinement. *R*-factors based on *F*^2^ are statistically about twice as large as those based on *F*, and *R*- factors based on ALL data will be even larger.

### Fractional atomic coordinates and isotropic or equivalent isotropic displacement parameters (Å^2^)

**Table:**

	*x*	*y*	*z*	*U*_iso_*/*U*_eq_	
C1	1.3188 (5)	1.3283 (5)	1.4807 (3)	0.0753 (11)	
H1A	1.3746	1.2709	1.4639	0.113*	
H1B	1.3938	1.4078	1.5421	0.113*	
H1C	1.2897	1.3696	1.4264	0.113*	
C2	1.0102 (4)	1.0784 (3)	1.3801 (2)	0.0407 (6)	
C3	0.9324 (3)	0.8957 (3)	1.2084 (2)	0.0391 (6)	
C4	0.9441 (3)	0.8476 (3)	1.1199 (2)	0.0385 (6)	
C5	1.0573 (4)	0.8202 (3)	0.9740 (2)	0.0390 (6)	
C6	1.1888 (4)	0.8549 (3)	0.9252 (2)	0.0468 (7)	
H6A	1.2329	0.9591	0.9262	0.056*	
H6B	1.1347	0.7950	0.8548	0.056*	
C7	1.2681 (5)	0.6217 (4)	0.9544 (3)	0.0651 (9)	
H7A	1.3327	0.5927	1.0056	0.078*	
H7B	1.1542	0.5855	0.9562	0.078*	
C8	1.2573 (7)	0.5487 (5)	0.8545 (4)	0.0880 (13)	
H8A	1.3674	0.5934	0.8475	0.106*	
H8B	1.2227	0.4433	0.8467	0.106*	
C9	1.0860 (8)	0.4660 (7)	0.6849 (4)	0.1128 (19)	
H9A	1.1787	0.4945	0.6595	0.135*	
H9B	1.0565	0.3662	0.6920	0.135*	
C10	0.9459 (9)	0.4676 (9)	0.6175 (4)	0.131 (3)	
H10A	0.8974	0.3828	0.5580	0.157*	
H10B	0.9870	0.5570	0.5958	0.157*	
C11	0.7180 (7)	0.3431 (5)	0.6827 (4)	0.0914 (14)	
H11A	0.7741	0.2801	0.6902	0.110*	
H11B	0.6044	0.2836	0.6321	0.110*	
C12	0.7074 (4)	0.4011 (4)	0.7818 (3)	0.0623 (9)	
H12A	0.8214	0.4560	0.8325	0.075*	
H12B	0.6441	0.3173	0.8033	0.075*	
C13	0.7897 (4)	0.7058 (4)	0.8160 (2)	0.0529 (7)	
H13A	0.8643	0.7022	0.7798	0.063*	
H13B	0.7503	0.7771	0.7975	0.063*	
C14	0.8897 (4)	0.7596 (3)	0.9285 (2)	0.0396 (6)	
C15	0.8397 (4)	1.0198 (3)	1.3351 (2)	0.0443 (6)	
C16	0.5893 (6)	1.1099 (6)	1.2868 (4)	0.0912 (14)	
H16A	0.6654	1.1911	1.2698	0.137*	
H16B	0.5123	1.1380	1.3080	0.137*	
H16C	0.5258	1.0238	1.2283	0.137*	
O1	1.1364 (5)	0.5674 (4)	0.7799 (2)	0.0980 (10)	
O2	0.8095 (6)	0.4633 (5)	0.6516 (3)	0.1180 (14)	
S1	1.12983 (11)	1.21034 (9)	1.49854 (6)	0.0562 (2)	
S2	1.10990 (9)	0.99321 (8)	1.32208 (5)	0.0452 (2)	
S3	1.13813 (9)	0.87356 (9)	1.10784 (5)	0.0456 (2)	
S4	1.36459 (10)	0.82043 (9)	0.98642 (7)	0.0545 (2)	
S5	0.60655 (10)	0.52141 (11)	0.77587 (7)	0.0664 (3)	
S6	0.76769 (9)	0.74362 (8)	1.00707 (5)	0.0456 (2)	
S7	0.74108 (9)	0.86596 (9)	1.22600 (6)	0.0486 (2)	
S8	0.71029 (12)	1.06618 (12)	1.38866 (7)	0.0675 (3)	

### Atomic displacement parameters (Å^2^)

**Table:**

	*U*^11^	*U*^22^	*U*^33^	*U*^12^	*U*^13^	*U*^23^
C1	0.062 (2)	0.072 (3)	0.051 (2)	0.0072 (19)	0.0037 (16)	−0.0007 (18)
C2	0.0527 (16)	0.0355 (14)	0.0323 (14)	0.0190 (12)	0.0161 (12)	0.0072 (11)
C3	0.0420 (14)	0.0340 (14)	0.0362 (14)	0.0157 (12)	0.0118 (11)	0.0049 (11)
C4	0.0411 (14)	0.0338 (14)	0.0360 (14)	0.0174 (11)	0.0103 (11)	0.0037 (11)
C5	0.0522 (16)	0.0361 (14)	0.0335 (14)	0.0279 (13)	0.0129 (12)	0.0057 (11)
C6	0.0524 (16)	0.0505 (17)	0.0488 (17)	0.0315 (14)	0.0221 (13)	0.0149 (14)
C7	0.082 (2)	0.054 (2)	0.076 (2)	0.0400 (19)	0.037 (2)	0.0251 (18)
C8	0.121 (4)	0.068 (3)	0.091 (3)	0.057 (3)	0.046 (3)	0.013 (2)
C9	0.122 (4)	0.122 (4)	0.080 (3)	0.051 (4)	0.046 (3)	−0.016 (3)
C10	0.187 (6)	0.230 (8)	0.069 (3)	0.164 (6)	0.076 (4)	0.046 (4)
C11	0.117 (4)	0.077 (3)	0.089 (3)	0.063 (3)	0.030 (3)	0.008 (2)
C12	0.0570 (19)	0.054 (2)	0.062 (2)	0.0141 (16)	0.0183 (16)	0.0165 (17)
C13	0.0618 (18)	0.0575 (19)	0.0353 (16)	0.0327 (16)	0.0074 (13)	0.0055 (14)
C14	0.0506 (15)	0.0340 (14)	0.0346 (14)	0.0240 (12)	0.0116 (12)	0.0046 (11)
C15	0.0539 (16)	0.0419 (16)	0.0398 (15)	0.0208 (13)	0.0233 (13)	0.0099 (12)
C16	0.073 (3)	0.117 (4)	0.094 (3)	0.062 (3)	0.026 (2)	0.014 (3)
O1	0.123 (3)	0.077 (2)	0.0679 (19)	0.0270 (19)	0.0434 (18)	−0.0154 (15)
O2	0.174 (4)	0.173 (4)	0.125 (3)	0.139 (3)	0.107 (3)	0.092 (3)
S1	0.0735 (5)	0.0488 (5)	0.0330 (4)	0.0198 (4)	0.0176 (4)	0.0014 (3)
S2	0.0465 (4)	0.0487 (4)	0.0353 (4)	0.0234 (3)	0.0099 (3)	0.0029 (3)
S3	0.0433 (4)	0.0557 (5)	0.0351 (4)	0.0277 (3)	0.0082 (3)	0.0015 (3)
S4	0.0418 (4)	0.0531 (5)	0.0658 (5)	0.0231 (4)	0.0160 (4)	0.0109 (4)
S5	0.0432 (4)	0.0805 (6)	0.0507 (5)	0.0237 (4)	0.0052 (3)	−0.0148 (4)
S6	0.0404 (4)	0.0461 (4)	0.0384 (4)	0.0164 (3)	0.0089 (3)	−0.0005 (3)
S7	0.0433 (4)	0.0459 (4)	0.0454 (4)	0.0121 (3)	0.0176 (3)	0.0029 (3)
S8	0.0690 (5)	0.0832 (7)	0.0609 (6)	0.0388 (5)	0.0379 (4)	0.0115 (5)

### Geometric parameters (Å, °)

**Table:**

C1—S1	1.786 (4)	C9—C10	1.391 (8)
C1—H1A	0.9600	C9—O1	1.410 (5)
C1—H1B	0.9600	C9—H9A	0.9700
C1—H1C	0.9600	C9—H9B	0.9700
C2—C15	1.351 (4)	C10—O2	1.448 (6)
C2—S1	1.741 (3)	C10—H10A	0.9700
C2—S2	1.760 (3)	C10—H10B	0.9700
C3—C4	1.335 (4)	C11—O2	1.394 (6)
C3—S7	1.753 (3)	C11—C12	1.493 (6)
C3—S2	1.759 (3)	C11—H11A	0.9700
C4—S6	1.753 (3)	C11—H11B	0.9700
C4—S3	1.753 (3)	C12—S5	1.802 (4)
C5—C14	1.327 (4)	C12—H12A	0.9700
C5—C6	1.500 (4)	C12—H12B	0.9700
C5—S3	1.763 (3)	C13—C14	1.506 (4)
C6—S4	1.809 (3)	C13—S5	1.828 (4)
C6—H6A	0.9700	C13—H13A	0.9700
C6—H6B	0.9700	C13—H13B	0.9700
C7—C8	1.465 (6)	C14—S6	1.767 (3)
C7—S4	1.782 (4)	C15—S8	1.744 (3)
C7—H7A	0.9700	C15—S7	1.760 (3)
C7—H7B	0.9700	C16—S8	1.806 (5)
C8—O1	1.411 (6)	C16—H16A	0.9600
C8—H8A	0.9700	C16—H16B	0.9600
C8—H8B	0.9700	C16—H16C	0.9600
			
S1—C1—H1A	109.5	O2—C10—H10A	107.7
S1—C1—H1B	109.5	C9—C10—H10B	107.7
H1A—C1—H1B	109.5	O2—C10—H10B	107.7
S1—C1—H1C	109.5	H10A—C10—H10B	107.1
H1A—C1—H1C	109.5	O2—C11—C12	109.1 (4)
H1B—C1—H1C	109.5	O2—C11—H11A	109.9
C15—C2—S1	124.6 (2)	C12—C11—H11A	109.9
C15—C2—S2	116.2 (2)	O2—C11—H11B	109.9
S1—C2—S2	118.48 (17)	C12—C11—H11B	109.9
C4—C3—S7	124.5 (2)	H11A—C11—H11B	108.3
C4—C3—S2	123.0 (2)	C11—C12—S5	113.1 (3)
S7—C3—S2	112.44 (15)	C11—C12—H12A	109.0
C3—C4—S6	123.6 (2)	S5—C12—H12A	108.9
C3—C4—S3	122.6 (2)	C11—C12—H12B	108.9
S6—C4—S3	113.75 (15)	S5—C12—H12B	109.0
C14—C5—C6	127.7 (3)	H12A—C12—H12B	107.8
C14—C5—S3	116.9 (2)	C14—C13—S5	113.2 (2)
C6—C5—S3	115.3 (2)	C14—C13—H13A	108.9
C5—C6—S4	113.8 (2)	S5—C13—H13A	108.9
C5—C6—H6A	108.8	C14—C13—H13B	108.9
S4—C6—H6A	108.8	S5—C13—H13B	108.9
C5—C6—H6B	108.8	H13A—C13—H13B	107.7
S4—C6—H6B	108.8	C5—C14—C13	127.3 (3)
H6A—C6—H6B	107.7	C5—C14—S6	117.3 (2)
C8—C7—S4	114.3 (3)	C13—C14—S6	115.4 (2)
C8—C7—H7A	108.7	C2—C15—S8	124.2 (2)
S4—C7—H7A	108.7	C2—C15—S7	116.8 (2)
C8—C7—H7B	108.7	S8—C15—S7	118.15 (17)
S4—C7—H7B	108.7	S8—C16—H16A	109.5
H7A—C7—H7B	107.6	S8—C16—H16B	109.5
O1—C8—C7	108.0 (3)	H16A—C16—H16B	109.5
O1—C8—H8A	110.1	S8—C16—H16C	109.5
C7—C8—H8A	110.1	H16A—C16—H16C	109.5
O1—C8—H8B	110.1	H16B—C16—H16C	109.5
C7—C8—H8B	110.1	C9—O1—C8	110.0 (4)
H8A—C8—H8B	108.4	C11—O2—C10	121.3 (4)
C10—C9—O1	108.7 (5)	C2—S1—C1	102.60 (16)
C10—C9—H9A	109.9	C3—S2—C2	94.09 (13)
O1—C9—H9A	109.9	C4—S3—C5	94.92 (13)
C10—C9—H9B	109.9	C7—S4—C6	102.45 (18)
O1—C9—H9B	109.9	C12—S5—C13	101.68 (16)
H9A—C9—H9B	108.3	C4—S6—C14	94.74 (13)
C9—C10—O2	118.5 (4)	C3—S7—C15	93.90 (14)
C9—C10—H10A	107.7	C15—S8—C16	101.88 (18)

### Hydrogen-bond geometry (Å, °)

**Table:**

*D*—H···*A*	*D*—H	H···*A*	*D*···*A*	*D*—H···*A*
C8—H8A···S5^i^	0.97	2.94	3.762 (5)	143
C1—H1C···O2^ii^	0.96	2.49	3.379 (6)	154

Symmetry codes: (i) *x*+1, *y*, *z*; (ii) −*x*+2, −*y*+2, −*z*+2.
